# Cancer-related circular RNA: diverse biological functions

**DOI:** 10.1186/s12935-020-01703-z

**Published:** 2021-01-06

**Authors:** Dan Cheng, Jing Wang, Zigang Dong, Xiang Li

**Affiliations:** 1 Department of Pathophysiology, School of Basic Medical Sciences, Zhengzhou University, Zhengzhou, 450001 Henan China; 2 China-US (Henan) Hormel Cancer Institute, No. 127, Dongming Road, Jinshui District, Zhengzhou, 450008 Henan China

**Keywords:** CircRNAs, Biogenesis, Back-splicing, miRNA sponge, Biomarker

## Abstract

Noncoding RNAs, including long noncoding RNAs (lncRNAs), microRNAs (miRNAs) and circular RNAs (circRNAs), are involved in regulating biological functions. In recent decades, miRNAs and lncRNAs have both inspired a wave of research, but the study of circRNA functions is still in its infancy. Studies have found that circRNAs actively participate in the occurrence and development of various diseases, which emphasizes the importance of circRNAs. Here, we review the features and classification of circRNAs and summarize their functions. Then, we briefly describe how to analyze circRNAs by bioinformatics procedures. In addition, the relationship between circRNAs and cancers is discussed with an emphasis on proving whether circRNAs can be potential biomarkers for the prognosis and diagnosis of cancer.

## Background

CircRNAs are a class of noncoding RNAs without 3′-poly(A) tails and 5′-caps that have closed circular structures formed by covalent bonding. They were first discovered in a plant virus by electron microscopy in 1976 [1], and then in 1979, Hsu et al. [2] provided evidence of the existence of circRNAs in several eukaryotic cells. In the following decades, although there were many discoveries about circRNAs, such as the first observation of circRNAs in humans [3] and the identification of four circRNAs expressed from the DCC (deleted in colorectal cancer) gene [4], the function of circRNAs had not come into great notice. Prior to 2013, circRNAs were regarded as a product of the incorrect splicing of pre-mRNA. Then, in the wake of the emergence of next-generation RNA sequencing (RNA-seq) and the development of bioinformatics, it was found that circRNAs are abundant and dynamically expressed in several eukaryotic cells, have high diversity and conservation, and play an irreplaceable role in the development of organisms [5]. Some publications have shown that circRNAs are closely related to cancers and can be involved in the regulation of tumorigenesis by adsorbing miRNAs, interacting with proteins, regulating the transcription of mRNA or translation into protein. However, more efforts are needed to explore the concrete functions of circRNAs in cancers.

## The characteristics and categories of CircRNAs

### Characteristics of circRNAs

In the human genome, RNAs that encode proteins make up only approximately 2% of the total sequence, and most of the remaining sequences are noncoding RNAs. Noncoding RNAs play an important role in regulating biological functions. CircRNAs, a member of the noncoding RNA family, possess some important features.

First, circRNAs have a special circular structure that protects them from degradation by RNase R or RNA exonucleases, which makes circRNAs more stable than their linear counterparts. In terms of stability, Jeck et al. [6] reported that the average half-life of circRNAs is approximately 5 times longer than that of mRNAs in most species.

Second, with the application of RNA sequencing technologies that are independent of poly(A) purification [7], several studies have reported that circRNAs are abundant in eukaryotic cells. More than 20,000 different circRNAs from eukaryotes have been identified [8], and the number will continue to grow. The study by Memczak et al. [9] identified 724 circRNAs in *C. elegans*, 1903 circRNAs in mice and 1950 circRNAs in humans. Evidence showed that circRNAs are widespread in human cancer genomes. There were 92,589 circRNAs identified across ~ 1000 cancer cell lines [10], and circRNAs were found in more than 800 tumor samples [11]. Additionally, some circRNAs have much higher expression than their liner mRNAs in human cells [5, 12].

Third, circRNAs exhibit sequence conservation among species to some extent [13]. For instance, it has been reported that 4522 out of 15,849 circRNAs in mice possess homologous sequences in humans [14]. This feature proves that circRNAs are not the outcome of aberrant splicing, as was previously thought.

Moreover, You et al. [15] observed that circRNAs originating from Rmst and Klhl2 are much more highly expressed in the mouse brain than in the liver or lung. Moreover, according to the sequence data, some nematode circRNAs are not expressed in 1- or 2-cell embryos but seem to exist in oocytes [9]. In general, these discoveries indicate that circRNAs are expressed in specific tissues and cells.

### Biogenesis of circRNAs

**Fig. 1:**
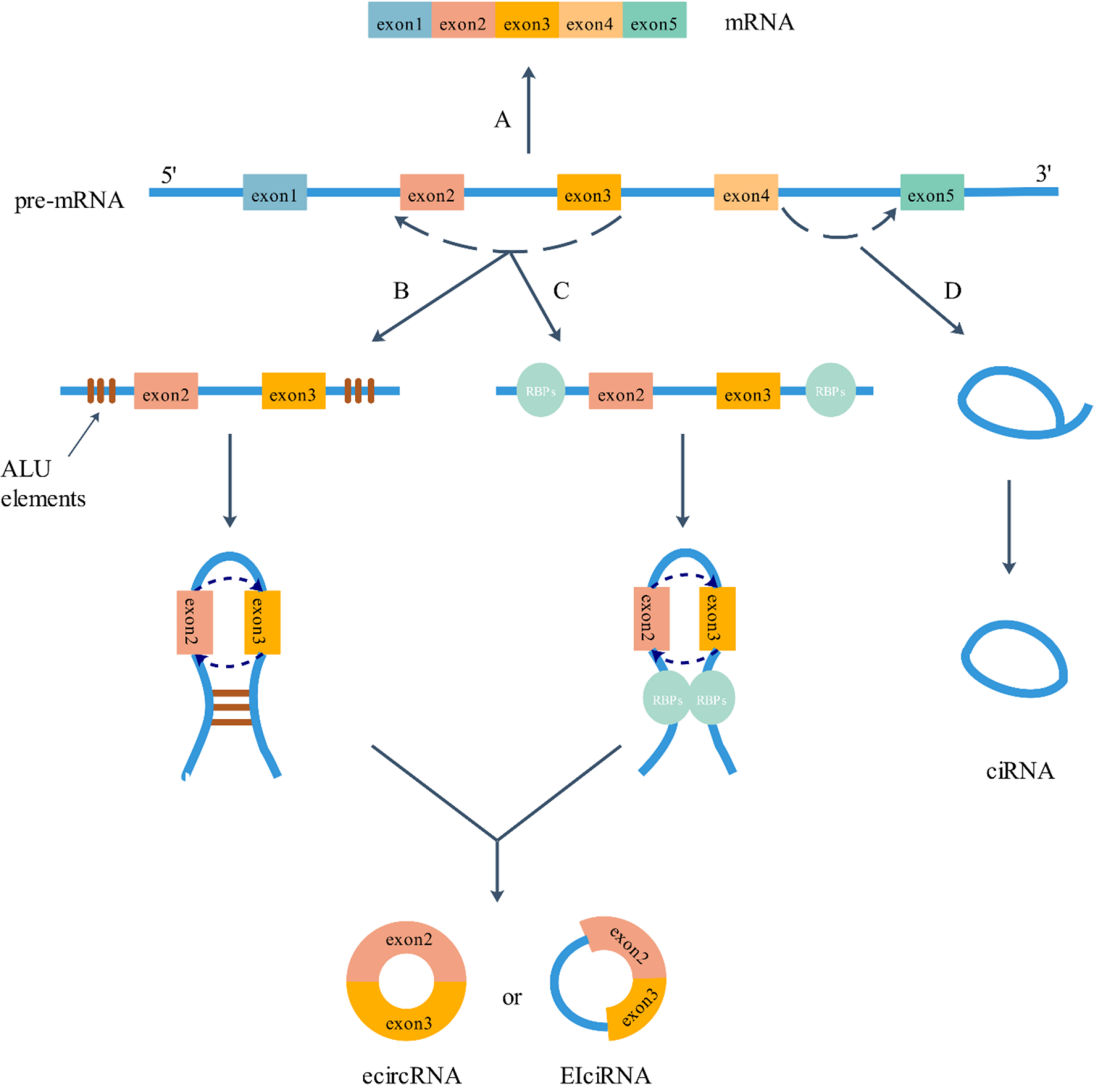
Biogenesis of CircRNAs. A: Canonical splicing. A process that removes the introns of pre-mRNAs, and then exons are connected to be a liner mRNA. B, C: Backing splicing. intron pairing and RNA binding proteins (RBPs) that can promote the circularization of back splicing to generate ecircRNAs or EIcicRNA. D: Intron splicing. Lariat introns that failure to debranch at the branch point site and trimming of the lariat tail can produce ciRNAs via intron splicing

It is crucial to reveal how circRNAs are generated to establish their classification. Canonical splicing, with activation of spliceosomal machinery, is a process that removes the introns of precursor messenger RNAs (pre-mRNAs), after which exons are connected to form liner mRNAs [16] (Fig. 1a). However, unlike the canonical splicing of mRNAs, circRNAs originate from spliceosome-mediated nonsequential back-splicing of pre-mRNAs [17]. In the process of back-splicing, the upstream 5′ splice donor site is reversibly paired to the downstream 3′ splice acceptor site to generate a covalently closed structure [17, 18]. Recent studies have shown that canonical splicing signals and canonical splicing machinery are both indispensable for back-splicing [19, 20].

Two main factors, intron pairing and RNA binding proteins (RBPs), can promote back-splicing circularization. On the one hand, intron pairing induces exons to approach each other by ALU elements [21] or nonrepetitive but complementary sequences [22] (Fig. 1b). For the formation of circHIPK3, long flanking introns that possess complementary ALU repeats are required [23]. On the other hand, some RBPs, such as MBL (MBNL1), QKI and FUS [24], can bind strongly and specifically to conserved sequences of flanking introns to promote exon circularization, and finally facilitating the formation of circRNAs [20] (Fig. 1c). In addition, the effect of double-stranded RNA (dsRNA) specific adenosine deaminase (ADAR) on the editing of adenosine to inosine [25] and the unwinding of dsRNA helix structure by ATP-dependent RNA helicase A (also known as DHX9) [26, 27] suppress the biogenesis of circRNAs.

Furthermore, circRNA generation can be facilitated by splicing factors (SFs) like ESRP1 [28] and the elongation velocity of RNA polymerase II [29].

### Categories of circRNAs

CircRNAs are divided into three main classes based on their different structures: circRNA from exons (ecircRNA), circRNA with 3′,5′- or 2′,5′- phosphodiester bond (ciRNA) and circRNA from exon–intron junctions (EIciRNA).

EcircRNAs are the largest subclass of circRNAs, accounting for approximately 85% of circRNAs [9]. EcircRNAs include only exons that can be one or more, but most ecircRNAs span less than 5 exons [30]. Some researchers have found that ecircRNAs are mainly located in the cytoplasm [6]. The biogenesis of ecircRNAs consists of two mechanisms: direct back-splicing and exon skipping [5, 17, 31]. During the process of direct back-splicing, alternatively spliced RNA and a lariat intermediate that includes exons are first formed, after which canonical splicing removes the introns of the lariat to generate ecircRNA transcripts [12, 32, 33]. Comparatively speaking, exon skipping is the process of a downstream exon jumping over one or several exons to connect to an upstream exon, thus forming a circRNA after removal of the introns [5, 34, 35]. Recent reports have proven that direct back-splicing may be the main mechanism to form ecircRNAs [6]. EcircRNAs can interact with miRNA RBPs or can be translated into proteins to play a role in gene expression regulation [36].

CiRNAs, which with 3′,5′- or 2′,5′- phosphodiester bond and mainly exist in the nucleus, are involved in the regulation of their parental mRNA [37] and increase the expression of parental genes [36]. During conventional splicing intron lariats excised can sometimes escape debranching and retain a circular form with a 3′,5′- or 2′,5′-phosphodiester bond between the splice donor and the branch point [38] (Fig. 1d).

Composed of both exons and introns, EIciRNAs share some features with both ecircRNAs and ciRNAs [36]. Similar to ciRNAs, EIciRNAs are also predominantly located in the nucleus and regulate the expression of their parental gene in the nucleus [39, 40]. The biogenesis of EIciRNAs is similar to that of ecircRNAs; however, while ecircRNAs are generated when the introns in the lariat are completely removed, EIciRNAs are produced when the introns are retained.

### Functions

Although circRNAs were discovered a long time ago, research on their functions is still in its infancy. The different distributions of circRNAs exhibit distinctive functions: circRNAs located in the cytoplasm mainly function as miRNA sponges and have the potential to be translated, while circRNAs scattered in the nucleus may regulate the transcription of parental genes.

### Acting as miRNA sponges

**Fig. 2:**
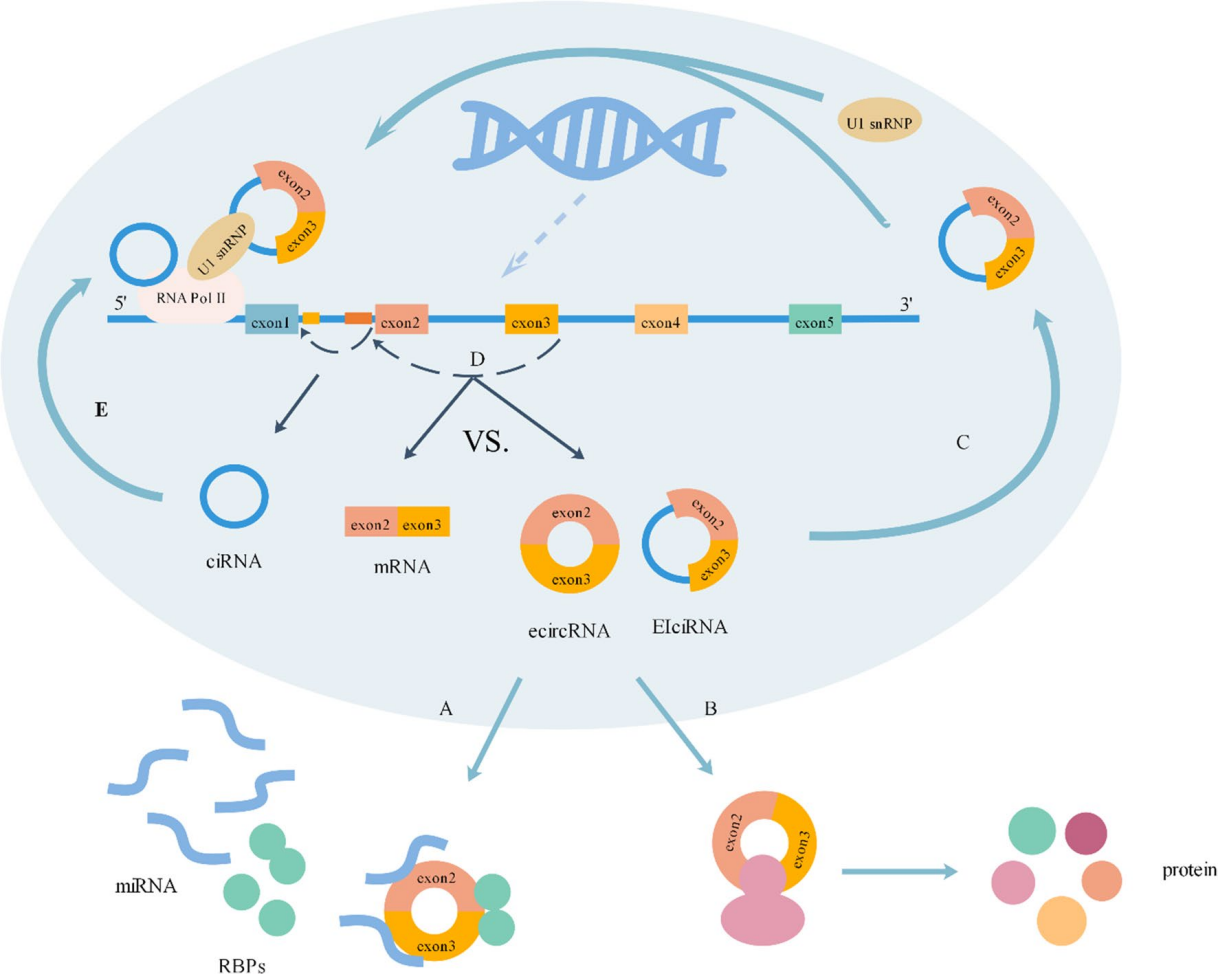
Functions of CirxRNAs. A: Regulating transcription. CiRNAs which mainly located in the nucleus can bind to RNA polymerase II at the promoters of their host genes to enhance gene expression. B :Regulating alternative splicing. Circularization and splicing compete against each other, the formation of circRNAs impact the alternative splicing of pre-mRNAs, eventually giving rise to alteration of gene expression. C: Regulating transcription. EIciRNAs can interact with U1 snRNP and then the EIciRNAs-U1 snRNP complexes associate with RNA polymerase II (Pol II) at the promoters of their host genes to enhance gene expression. D: Acting as sponges. EcircRNAs can bind to miRNAs and proteins to regulate gene expression. E: Translating into proteins. Some ecircRNAs can be encoded to proteins, thereby influence their downstreams

As a pivotal regulator of gene expression [41], miRNA can bind to mRNA by complementary sequences in the 3′ UTR of target mRNA, thereby inhibiting mRNA translation or promoting mRNA degradation. Some scientific studies have reported that ecircRNAs distributed in the cytoplasm contain miRNA response elements (MREs) that can absorb miRNA and prevent them from binding to their target mRNAs [42] (Fig. 2a). circRNAs can bind to one miRNA with several binding sites or bind to multiple miRNAs. CDR1as, cerebellar degeneration-related protein 1 antisense, harbors over 70 conventional miR-7 binding sites; therefore, it can regulate the expression of target mRNAs by absorbing miR-7 [43].

circHIPK3 [44] can sponge to 9 miRNAs with 18 potential binding sites and circCCDC66 [45] was potentially targeted by 99 miRNAs. Another example of circRNA-miRNA interactions has been shown for circ-FOXP1. A recent report revealed that circ-FOXP1 controlling mesenchymal stem cell (MSC) identity and differentiation was enriched in MSCs. Circ-FOXP1 can act as a miRNA sponge targeting miR-17–3p and miR-127–5p and promotes the proliferation and differentiation of MSCs [46].

### Translating into proteins

Although circRNAs are regarded as a class of noncoding RNAs, increasing evidence has demonstrated that some circRNAs are translatable [4] (Fig. 2b). Unlike mature mRNA translation, which normally requires a 5′ end 7-methylguanosine (m^7^G) cap structure and a 3′ poly(A) tail [47], circRNAs have different translation processes because they do not have these necessary structures. In 1998, it was confirmed that a circular mRNA containing a complete open reading frame (ORF) of green fluorescent protein (GFP) could successfully encode GFP in *Escherichia coli* [48]. A subsequent study showed that some circRNAs possessing internal ribosome entry sites (IRESs) can interact with ribosomes as the entry point, thus initiating translation [17, 41]. Moreover, surprisingly, it was found that N^6^-methyladenosine (m^6^A), the most common RNA modification, facilitates the protein-coding ability of circRNAs [49]. A recent study documented that FBXW7-185aa, a novel 21-kDa protein, was encoded by a spanning junction open reading frame in circ-FBXW7 driven by an internal ribosome entry site. Upregulation of FBXW7-185aa inhibited proliferation and cell cycle progression and reduced the half-life of c-Myc by antagonizing USP28-induced c-Myc stabilization in glioblastoma, while knockdown of FBXW7-185aa promoted malignant phenotypes in vitro and in vivo [50]. The protein-coding function provides a fresh viewpoint on the role of circRNAs in diseases.

### Regulating transcription

Due to being predominantly located in the nucleus and containing a relatively low number of microRNA target sites, EIciRNAs and ciRNAs have been found to be involved in transcription regulation [4, 47]. Some studies have suggested that these circRNAs modulate the expression of their parental genes [51]. A recent study indicated that the intron of EIciRNAs has one putative U1 snRNP (small nuclear ribonucleoprotein) binding site [36]; therefore, EIciRNAs can interact with U1 snRNP, and then the EIciRNAs-U1 snRNP complexes associate with RNA polymerase II (Pol II) at the promoters of their host genes to enhance gene expression [40] (Fig. 2c). For ciRNAs, it has been reported that ci-ankrd52 and ci-sirt7 bind to the extended Pol II complex at the transcriptional start site and positively regulate Pol II-mediated transcription, suggesting that they may also have cis-regulatory effects on upstream genes [4, 37] (Fig. 2e). The interplay between circRNAs and the transcriptional machinery provides new perspectives for strategies regulating gene expression in cells [52].

### Regulating alternative splicing

Circularization and splicing compete against each other, enabling ecircRNAs to function in alternative splicing [20] (Fig. 2d). When the formation of circRNA and liner mRNA involves the same exon, competition occurs, and the more exons are circularized, the less the same exons will appear in the processed mRNA [4, 53]. Therefore, the formation of circRNAs impacts the alternative splicing of pre-mRNAs, eventually resulting in altered gene expression. This is best illustrated by the example of circMbl, which is circularized from the second exon of the splicing factor *muscleblind* (MBL) gene, whereby modulation of MBL levels strongly affects the biosynthesis of circMbl [20].

### Binding to proteins

In addition to interacting with miRNAs, circRNAs can bind to proteins to regulate gene expression and ultimately influence the development of some diseases (Fig. 2a). For example, circ-Foxo3 can interact with the stress-related proteins FAK and HIF-1α in the cytoplasm and then block these proteins from entering the nucleus, which ultimately promotes cardiac senescence [54]. In contrast, Yang et al. revealed that circ-Amotl1 can interact with Stat3 and facilitate Stat3 nuclear translocation. By binding to the Stat3 and Dnmt3a promoters, circ-Amotl1 promotes Dnmt3a transcription, and the increased Dnmt3a can methylate the miR-17 gene promoter to inhibit the expression of miR-17-5p but increase fibronectin expression, which leads to accelerated wound repair [55].

**Fig. 3:**
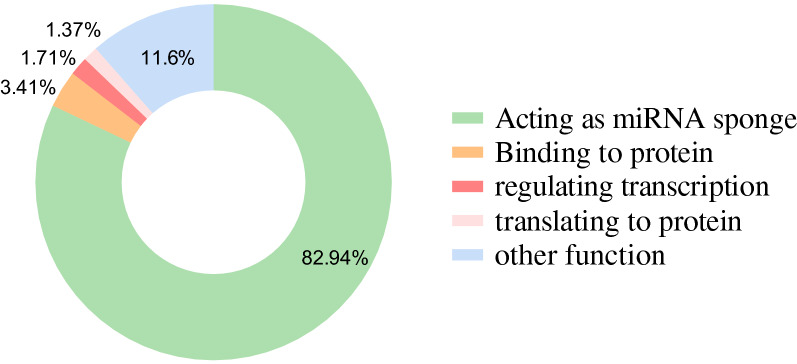
The proportion of circRNAs with different functions in cancers

Although circRNAs have different functions, they mainly adsorb miRNAs and then affect downstream targets involved in the development of cancers; the other functions account for only a small portion (Fig. 3). Table 1 shows the proportion of circRNAs with different functions in different tumors. Therefore, efforts need to be made to explore other functions of circRNAs in tumors.

**Table 1:** The proportion of circRNAs with different functions in cancers

The types of cancers	Acting as miRNA sponge	Regulating transcription	Binding to protein	Translating to protein	Other function
Hepatocellular carcinoma	79.6% (39/49)	2.0% (1/49)	4.1% (2/49)	2.0% (1/49)	12.2% (6/49)
Lung cancer	79.4% (27/34)	2.9% (1/34)	/	/	17.6% (6/34)
Breast cancer	87.9% (29/33)	/	3.0% (1/33)	/	9.1% (3/33)
Gastric cancer	84.8% (28/33)	3.0% (1/33)	6.1% (2/33)	/	9.1% (3/33)
Glioma	82.6% (19/23)	/	4.3% (1/23)	8.7% (2/23)	4.3% (1/23)
Colorectal cancer	69.6% (16/23)	4.3% (1/23)	8.7% (2/23)	/	17.4% (4/23)
Bladder cancer	94.7% (18/19)	/	/	/	5.3% (1/19)
Osteosarcoma	88.2% (15/17)	5.9% (1/17)	5.9% (1/17)	/	5.9% (1/17)
Cervical cancer	88.9% (8/9)	/	/	/	11.1% (1/9)
Oral squamous cell carcinomas	55.6% (5/9)	/	/	/	44.4% (4/9)
Leukemia	85.7% (6/7)	/	14.3% (1/7)	/	/
Esophageal squamous cell carcinoma	71.4% (5/7)	/	/	/	28.6% (2/7)
Pancreatic cancer	100.0% (6/6)	/	/	/	/
Pancreatic ductal adenocarcinoma	100.0% (6/6)	/	/	/	/
Glioblastoma	66.7% (4/6)	/	/	16.7% (1/6)	16.7% (1/6)
Prostate cancer	75.0% (3/4)	/	/	/	25.0% (1/4)
Nasopharyngeal carcinoma	100.0% (3/3)	/	/	/	/
Renal carcinoma	100.0% (3/3)	/	/	/	/
Papillary thyroid carcinoma	100.0% (2/2)	/	/	/	/
Cutaneous squamous cell carcinoma	100.0% (1/1)	/	/	/	/

### CircRNAs are closely related to cancers

It has been reported that circRNAs play an important role in cancer progression by modulating many of the hallmarks of cancer, such as circRNAs being involved in the regulation of sustained proliferative signaling [56, 57], evasion of growth suppressors [58], impairment of differentiation signals, contribution to tumor metastasis and invasion [59], and induction of angiogenesis [60]. In addition, increasing reports have shown that circRNAs can serve as tumor promoters or suppressors to regulate signaling pathways of cancer, including the Wnt/β-catenin signaling [61], MAPK/ERK and PTEN/PIK3/AKT pathways [62]. Meanwhile, some important molecules such as p53 [63] and K-Ras [64] participate in the regulation of circRNAs.

Recently, the relationship between circRNAs and tumor microenvironment (TME) has raised some concern due to its influence on tumor immunity, angiogenesis, metastasis, and hypoxia. CircRNAs can modulate TME by mediating tumor immune surveillance, regulating immune escape via a circRNA-miRNA-PD-1/PD-L1 axis, and regulating the cytotoxicity of natural killer cells, thus promote or inhibit the immune system and angiogenesis, improve the permeability of endothelial cells, and remodel the extracellular matrix (ECM) [65].

**Fig. 4:**
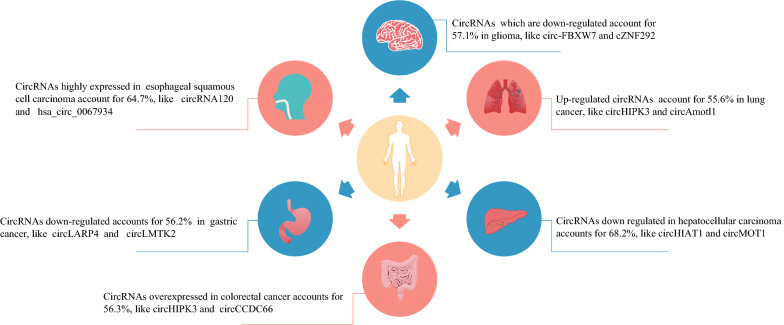
CircRNAs distribution in cancer. The figure above shows the presence of circRNAs in six major cancers

Herein, we will discuss the relationship between circRNAs and cancers (Fig. 4) and evaluate whether circRNAs can be biomarkers of the diagnosis and prognosis of cancer (see Table 2).

**Table 2:** Databases of circRNAs

Database	Websites	The date of launching	Function
circRNABase	http://starbase.sysu.edu.cn/starbase2/mirCircRNA.php	2013	Constructing interaction networks of miRNA and circRNA and circRNA and RBPs
Circ2Traits	http://gyanxet-beta.com/circdb/	2013	The first database to collect circRNAs potentially associated with human disease or traits
circBase	http://www.circbase.org/	2014	Information such as genome location, cell line or tissue source and references can be provided and downloaded for a specific circRNA
CIRCpedia	http://www.picb.ac.cn/rnomics/circpedia/	2016	Providing annotations of alternative splicing and back splicing events for circRNAs from different cell lines or tissues
deepBase v2.0	http://deepbase.sysu.edu.cn/	2016	A platform for analyzing and predicting the evolution, expression patterns and functions of ncRNA in 19 different species
CircInteractome	https://circinteractome.nia.nih.gov/	2016	Predicting potential binding sites for RBPs and circRNA, miRNAs and circRNA, designing primers and siRNA for circRNAs
CircNet	http://circnet.mbc.nctu.edu.tw/	2016	The first public information platform that provides tissue-specific circRNA expression and circRNA-miRNA-mRNA regulatory networks
CircRNADb	http://202.195.183.4:8000/circrnadb/circRNADb.php	2016	The first circRNA database with the ability to encode proteins, providing information on the translational potential of circRNA and its related proteins
TSCD	http://gb.whu.edu.cn/TSCD/	2017	Searching for tissue-specific circRNA information in humans and mouse and predicting bound miRNAs
exoRBase	http://www.exorbase.org/	2017	Containing mRNA, lncRNA and circRNA, from serum exosomal RNA-seq sequencing samples
CSCD	http://gb.whu.edu.cn/CSCD/	2018	Provides many tumor-specific circRNAs, predicting MRE, RBP binding sites, ORF, and analyzing alternative splicing of related genes
circlncRNAnet	http://app.cgu.edu.tw/circlnc/	2018	An online analysis database that integrates the functional network of lncRNA and circRNA, supporting online visual analysis
circRNA disease	http://cgga.org.cn:9091/circRNADisease/	2018	Collecting human disease-related circRNAs and searching by circRNA name or disease name is supported
CircR2Disease	http://bioinfo.snnu.edu.cn/CircR2Disease/	2018	Revealing the relationship between circRNA and disease, and building an interaction network of circRNA and diseases

### CircRNAs and hepatocellular cancer

Due to the aggressive nature and recurrence rate of hepatocellular cancer (HCC), the prognosis of HCC remains poor. Therefore, effective biomarkers are necessary to improve early diagnosis and prognosis analysis [66]. There is increasing evidence that circRNAs are related to the development and invasion of hepatoma, although the mechanism is not entirely clear. Liang et al. [67] reported that circβ-catenin was upregulated in liver cancer tissues compared to adjacent normal tissues. Circβ-catenin encodes a novel β-catenin isoform that promotes liver cancer growth and migration in vitro and attenuates tumorigenesis and metastasis in vivo by activating the Wnt pathway. A study by Song et al. [68] showed that circ0003998 was markedly upregulated in portal vein tumor thrombus (PVTT) metastasis and HCC tissues. Its expression was related to the aggressive characteristics of HCC patients. Circ0003998 promoted mesenchymal transition (EMT) in HCC by acting as a competing endogenous RNA (ceRNA) to sponge miR-143-3p to relieve the repressive effect on FOSL2 (EMT-related stimulator). Furthermore, circ0003998 could bind with PCBP1 to increase the expression level of the EMT-related gene CD44v6. In addition, Wang et al. [69] found that circHIAT1 was obviously downregulated in HCC samples and cell lines. The low expression of circHIAT1 was positively correlated with the poor overall survival of HCC patients. The overexpression of circHIAT1 suppressed HCC progression in vitro and in vivo by regulating the miR-3171/PTEN axis. Zheng et al. [70] found that hsa_circ_0079929 expression was lower in HCC tissues. The inhibitory effect of hsa_circ_0079299 was partially mediated by the PI3K/AKT/mTOR signaling pathway. Circ-0051443 was also strongly downregulated in plasma exosomes and tissues, and exosomal circ-0051443 could inhibit HCC progression by functioning as a sponge of miR-331-3p to promote BAK1 expression [71]. Circ-0051443 spread from normal cells to HCC cells through exosomes and inhibited malignant biological behaviors by promoting cell apoptosis and suppressing the cell cycle, indicating that exosomal circ-0051443 can serve as a predictor and potential therapeutic target for HCC.

### CircRNAs and breast cancer

CircRNAs are abnormally expressed in breast cancer. Chen et al. [72] performed high-throughput circular RNA microarray assays in 3 pairs of triple-negative breast cancer (TNBC) patient samples and identified TNBC-related circRNAs, of which 173 circRNAs were obviously upregulated, whereas 77 circRNAs were significantly downregulated. They found that circEPSTI1, derived from the EPSTI1 gene, was the most upregulated circRNA among the candidates. Due to the crucial role of the EPSTI1 gene in cancer invasion and metastasis, the authors focused their attention on circEPSTI1. They demonstrated that circEPSTI1 was highly expressed in most TNBC cell lines and tissues, and knockdown of circEPSTI1 reduced cell proliferation and induced apoptosis. The effects of circEPSTI1 could be explained by its interaction with miR-4753 and miR-6809, which then affected downstream BCL11A expression. In addition, high expression of circEPSTI1 was positively correlated with tumor size, lymph node infiltration and TNM stage, and TNBC patients with increased circEPSTI1 and BCL11A levels were significantly associated with reduced disease-free survival (DFS) and overall survival (OS), suggesting that circEPSTI1 is an independent prognostic marker of TNBC patient survival. Hsa_circ_0025202 was found to be downregulated in breast cancer cells and tissues and inversely correlated with lymphatic metastasis and histological grade, which reduced cell proliferation, colony formation, and migration and enhanced cell apoptosis and sensitized cells to tamoxifen (TAM) treatment [73]. In addition, hsa_circ_0025202 could absorb miR-182-5p to regulate the expression and activity of FOXO3a and then affect tumor inhibition and tamoxifen sensitization effects, indicating that hsa_circ_0025202 could be a therapeutic target in patients with HR-positive BC, especially in those receiving TAM therapy. In contrast to its host gene SMARCA5, circSMARCA5 is reduced in breast cancer tissues [74]. The enforced expression of circSMARCA5 could induce drug sensitivity in breast cancer cell lines in vitro and in vivo. There is a competitive relationship between the expression of circSMARCA5 and SMARCA5. circSMARCA5 can interact with its parental locus to form an R loop, which results in the suppression SMARCA5 exon 15 transcription. The expression of circSMARCA5 leads to the downregulation of SMARCA5 and the production of a truncated nonfunctional protein, while circSMARCA5 overexpression was sufficient to increase the sensitivity to cytotoxic drugs. The above evidence shows that circSMARCA5 may be used as a therapeutic target for patients with resistant breast cancer.

### CircRNAs and gastric cancer

Gastric cancer (GC) is the fifth most common cancer in the world with high morbidity and mortality [75]. With the deepening of research on circular RNAs, the relationship between circular RNAs and the diagnosis and prognosis of gastric cancer has received increasing attention. Ding et al. [76] used an online dataset to analyze overexpressed circRNAs in GC tissues compared to adjacent tissues and found that circ-DONSON was the most upregulated circRNA among the candidates. Circ-DONSON was proven to be highly expressed in GC tissues and cells. Additionally, the authors found that circ-DONSON expression was positively correlated with TNM stage and lymphoid metastasis. Higher expression of circ-DONSON in GC patients was correlated with a lower overall survival rate and disease-free survival rate, indicating that circ-DONSON might be a prognostic marker. In contrast to the miRNA sponge mechanism, circ-DONSON might regulate transcription due to its nuclear location. Ding et al. found that circ-DONSON could recruit the NURF complex to the SOX4 promoter and initiate its transcription, thereby regulating GC cell malignant behaviors. Zhang et al. [77] found that circNRIP1, upregulated in GC tissues, acted as a miRNA-149-5p sponge to promote GC progression via the AKT/mTOR pathway, and the expression level of circNRIP1 significantly correlated with GC tumor size and lymphatic invasion. Moreover, a new report showed that circPSMC3 was significantly downregulated in gastric cancer plasma, tissues and cells and associated with poor prognosis, whereby patients with lower circPSMC3 expression showed a reduced overall survival time [78]. CircPSMC3 could act as a ceRNA by sponging miR-296-5p to suppress the proliferation and metastasis of gastric cancer. CircLMTK2 expression was also significantly reduced in GC [79]. A lower level of circLMTK2 expression was correlated with decreased overall survival in GC patients, and a multivariate Cox hazards analysis showed that high circLMTK2 expression was an independent factor for OS, suggesting that circLMTK2 could be used as a prognostic factor in GC cases. Huang et al. [80] found that circAKT3 was upregulated in cisplatin (CDDP)-resistant GC tissues and cells compared with CDDP-sensitive samples, and the upregulation of circAKT3 in GC patients receiving CDDP therapy was significantly associated with aggressive characteristics and was an independent risk factor of disease-free survival. These results suggest that circRNAs may be novel biomarkers of the diagnosis and prognosis of gastric cancer and can be therapeutic targets.

### CircRNAs and lung cancer

Lung cancer is the leading cause of cancer-related death worldwide, and most cases present with advanced local infiltration and/or distant metastasis at diagnosis [81]. Despite ongoing efforts to improve the response to treatment and the discovery of lung cancer treatments that have shown survival benefits, the overall 5-year survival rate for advanced lung cancer remains below 15%, underscoring the need for early diagnosis and timely treatment [82]. Recent studies have shown that circRNAs may be involved in the development of lung cancer, which is beneficial to the diagnosis, prognosis and even treatment of lung cancer. Recently, Jin et al. [83] reported that the expression of circARHGAP10 was significantly increased in both non-small cell lung cancer tissues and cell lines, and a high level of circARHGAP10 expression was correlated with a poor prognosis. Knockdown of circARHGAP10 suppressed proliferation, metastasis and glycometabolism by targeting the miR-150-5p/GLUT1 axis in NSCLC. Thus, circARHGAP10 may be a potential target for NSCLC treatment. Similarly, circTP63, which was upregulated in lung squamous cell carcinoma (LUSC), was shown to be a sponge of miR-873-3p, thus abolishing the suppressive effect of miR-873-3p and increasing the level of FOXM1, which upregulated CENPA and CENPB, thereby facilitating cell cycle progression [84]. The upregulation of circTP63 was correlated with larger tumor size and higher TNM stage in LUSC patients, suggesting that circTP63 is associated with the prognosis of LUSC. More recently, Qin et al. [85] determined that circ-UBR5 was markedly reduced in NSCLC tissues and correlated with tumor differentiation. Surprisingly, circ-UBR5, a novel snRNA, could play a role in spliceosome-mediated RNA splicing regulation by binding to the splicing regulatory factors QKI and NOVA1 and U1 snRNA in the nucleus.

### CircRNAs and glioma

Glioma is the most common intracranial primary cancer with extraordinarily high morbidity and mortality worldwide [86, 87]. Although there are common methods for clinical treatment, such as surgery, radiotherapy, and chemotherapy, the long-term effects and postoperative outcomes for patients with glioma are still unsatisfactory [88, 89]. Recently, circSCAF11 was found to be significantly upregulated in glioma tissues and cell lines, and ectopic overexpression of circSCAF11 was closely correlated with the poor clinical outcome of glioma patients [90]. Mechanistic experiments further indicated that circSCAF11 accelerated glioma tumorigenesis through the miR-421/SP1/VEGFA axis, providing a potential target for circRNAs and glioma treatment. CircRNAs can not only act as a miRNA sponge but can also absorb some proteins to influence the progression of tumors. For instance, circCPA4, which is highly expressed in glioma tissues and positively related to poor outcome of glioma, could act as a sponge for let-7 to regulate the expression of CPA4 and glioma progression [91]. Therefore, circCPA4 could be a novel prognostic biomarker and target for glioma treatment. Furthermore, circRNAs have the ability to encode proteins involved in the development of glioma. Zhang et al. [92] found that circ-SHPRH used overlapping genetic codes to generate a 'UGA' stop codon, which resulted in the translation of the 17 kDa SHPRH-146aa, and the expression of circ-SHPRH and SHPRH-146aa was low in glioma. The overexpression of SHPRH-146aa could protect full-length SHPRH from degradation by the ubiquitin proteasome, and then stabilized SHPRH subsequently ubiquitinating proliferating cell nuclear antigen (PCNA) as an E3 ligase, leading to inhibited cell proliferation and tumorigenicity. These results suggest that circRNAs are involved in the development of glioma in different ways.

### CircRNAs and colorectal cancer

Xu et al. [93] performed secondary sequencing to profile circRNA expression in colorectal cancer (CRC) tissues and matched normal tissues. They found that in CRC tissues, 92 circRNAs were significantly upregulated, and 21 circRNAs were downregulated. In particular, the expression of circRNA_0001178 and circRNA_0000826 was increased in CRC tissues and showed potential diagnostic value with AUCs of 0.945 and 0.816, respectively. CircHIPK3 was also significantly upregulated in CRC tissues and cell lines, and ectopic expression of circHIPK3 effectively reversed the miR-7-induced attenuation of the malignant phenotype of CRC cells by increasing the expression level of miR-7 targeting proto-oncogenes (FAK, IGF1R, EGFR, YY1) [94]. Increased expression of circHIPK3 was significantly associated with tumor T status, lymph node metastasis, distant metastasis, and advanced clinical stage, and it was an independent prognostic factor of poor overall survival in CRC. These results indicate that circHIPK3 upregulation is an early event in CRC development and has a vital role in CRC progression. Li et al. [95] demonstrated that circITGA7 and its linear host gene ITGA7 were both significantly downregulated in colorectal cancer tissues and cell lines, and their decreased expression was correlated with CRC progression. CircITGA7 inhibited colorectal cancer growth and metastasis by modulating the Ras pathway and upregulating the transcription of its host gene ITGA7. Furthermore, the noncoding effects of circCCDC66 were demonstrated in a previous publication. CircCCDC66 expression was elevated in polyps, and all stages of colorectal cancer tissues and patients with a higher level of circCCDC66 had a poor prognosis, as evidenced by lower overall survival rates [45]. CircCCDC66 could exert its function by regulating various oncogenes, thus modulating multiple pathological processes, including cell proliferation, migration, invasion, and anchorage-independent growth.

Taken together, these findings highlight novel oncogenic and tumor suppressor functions of circRNAs in colon cancer progression and metastasis.

### CircRNAs and other tumors

The abnormal expression of circRNAs was also observed in other cancers, such as bladder cancer, osteosarcoma, esophageal squamous cell carcinoma and leukemia. CircSLC8A1 was downregulated in bladder cancer tissues and cell lines, and it could function as a tumor suppressor through interaction with miR-130b and miR-494 to inhibit bladder cancer progression by regulating PTEN [96]. CircLRP6 was significantly increased in osteosarcoma, and high expression of circLRP6 resulted in shorter disease-free survival and overall survival. CircLRP6 acted as an oncogene by binding to LSD1 and EZH2 to inhibit the expression of KLF2 and APC [97]. In addition, circRNA microarray analysis revealed that 698 circRNAs were differentially expressed in acute myeloid leukemia (AML) patients, with 282 circRNAs found to be upregulated and 416 to be downregulated [98].

Fan et al. [99] identified ESCC-related circRNAs and found that the expression of 1045 circRNAs was obviously upregulated, whereas that of 1032 circRNAs was significantly downregulated by using a circular RNA microarray analysis in 3 pairs of esophageal squamous cell carcinoma (ESCC) frozen tumor and nontumor tissues. Cao et al. [100] revealed that circRNA_100876 expression was significantly higher in ESCC tissues and cell lines, and its high expression was strongly correlated with tumor invasion depth, lymph node metastasis and vascular invasion, indicating that circRNA_100876 expression was linked to the clinical progression of ESCC and might represent a promising prognostic biomarker.

These findings indicate that circRNAs regulate multiple signaling pathways through multiple modes of action and are closely linked to tumors.

### Bioinformatic analysis of circRNAs

Although circRNAs have been studied for a long time, they are not easy to detect due to the previously inadequate technology. With the emergence of next-generation RNA sequencing, an increasing number of circRNAs have been identified. Herein, we summarize the research procedures to study circRNAs.

First, a cDNA library is constructed. The ribosomal rRNA in total RNA is first removed using a biotinylated specific probe. After purification, the RNA is fragmented with temperature and ionic environment. Subsequently, dNTPs are added to synthesize a cDNA strand, and then DNA polymerase I and RNase H are used to synthesize double-stranded cDNA. During cDNA double strand synthesis, the RNA template is removed, and dTTP is replaced by dUTP. The ligation product of the double-stranded cDNA product followed by the addition of the "A" base and the linker is amplified, and the final cDNA library is obtained after purification. Finally, the constructed sequencing library is sequenced.

Second, the raw data generated by sequencing are analyzed. In the first step, the low-quality, joint contamination, and unknown N content of the base are filtered out. The filtered data are called clean reads. In the second step, clean reads are mapped to the reference genome, and circRNAs are predicted by two software programs, CIRI and find_circ. In the third step, quantitative and differential expression analysis of circRNAs is performed after combining the results of the two software programs. The functional analysis of circRNAs mainly focuses on regulation of gene expression through interactions with multiple miRNAs, which can be analyzed by some databases like analysis of common targets (ACT) [101] and CircNet [102].

Then, experiments such as reverse transcription-PCR (RT-PCR), droplet digital PCR, northern blotting, and fluorescence in situ hybridization are conducted to verify the circRNAs [103]. To study the function of circRNAs, gene overexpression and knockdown are used to manipulate circRNA expression. For mechanistic studies and with reference to some databases, such as CircNet, circBase, Circ2Traits and circRNA Disease (see Table 3), researchers perform bioinformatic prediction, RNA immunoprecipitation, luciferase reporter assay, and RNA pull-down combined with mass spectrometry to reveal circRNA-miRNA and circRNA-protein interactions.

**Table 3:** Biomarker research of circRNAs in cancers

Cancer type	CircRNA	Existence	Expression	Clinical Value	Ref.
Gastric cancer	hsa_circRNA_102958	Tissue	Up	Diagnosis	[105]
	hsa_circ_0074362		Down		[106]
	hsa_circ_0003159		Down		[107]
	hsa_circ_0000190	Tissue and plasma	Down		[108]
	circPSMC3		Down		[78]
	circ-KIAA1244		Down		[109]
	hsa_circ_0000520		Down		[110]
	circ-DCAF6	Tissue	Up	Prognosis	[111]
	circPDSS1		Up		[112]
	circ-DONSON		Up		[76]
	circLMTK2		Down		[112]
	circLARP4		Down		[113]
	hsa_circ_0001895		Down	Diagnosis and prognosis	[114]
	hsa_circ_0065149	Plasma exosome	Down		[115]
Colorectal cancer	circRNA_0001178	Tissue	Up	Diagnosis	[93]
	circRNA_0000826		Up		[93]
	circVAPA	Tissue and plasma	Up		[116]
	hsa_circ_0004585	Tissue and peripheral blood	Up		[117]
	circDDX17	Tissue	Down		[118]
	circRNA0003906		Down		[119]
	hsa_circ_001988		Down		[120]
	circ-ITGA7		Down		[90]
	circHIPK3		Up	Prognosis	[89]
	circCCDC66		Up		[45]
	circ_0026344		Down		[121]
Lung cancer	circRNA_102231	Tissue	Up	Diagnosis	[122]
	hsa_circ_0014130		Up		[123]
	hsa_circ_0001946		Down		[124]
	hsa_circ_0033155		Down		[125]
	circ_0067934		Up	Prognosis	[126]
	hsa_circ_0000792		Up		[127]
	hsa_circRNA_103809		Up		[128]
	circARHGAP10		Up		[83]
	circTP63		Up		[84]
	circRNA_100876		Up		[129]
Hepatoma	circFBLIM1	Tissue	Up	Diagnosis	[63]
	circRNA_100338		Up		[54]
	hsa_circ_0005075		Up		[61]
	hsa_circ_0091579		Up	Diagnosis and prognosis	[130]
	hsa_circ_0128298		Up		[131]
	hsa_circ_0078602		Down		[132]
	circ-0051443	Plasma exosomes and tissues	Down	Diagnosis	[71]
Breast cancer	circGFRA1	Tissue	Up	Diagnosis	[133]
	hsa_circ_0072309		Up	Prognosis	[134]
	circKIF4A		Up		[135]
	CircEPSTI1		Up		[72]
	circ-ITCH		Down		[136]
	circKDM4C		Down		[137]
Bladder cancer	hsa_circ_0018069	Tissue	Down	Diagnosis	[138]
	circPRMT5	Tissue serum and urine exosome	Up	Prognosis	[139]
	circLPAR1	Tissue	Down		[140]
	circMTO1		Down		[141]
	hsa_circ_0000285	Tissue and serum	Down		[142]
Osteosarcoma	circPVT1	Tissue and serum	Up	Diagnosis	[143]
	circ_HIPK3	Tissue	Down		[144]
	hsa_circ_0002052		Down	Prognosis	[145]
	hsa_circ_0081001	Tissue and serum	Up	Diagnosis and prognosis	[146]
Esophageal squamous cell carcinoma	hsa_circ_0006168	Tissue	Up	Diagnosis	[147]
	cicrRNA_100876		Up	Prognosis	[100]
	hsa_circ_0067934		Up	PROGNOSIS	[148]
Leukemia	hsa_circ_0004277	Tissue	Down	Diagnosis	[149]
	circ-CBFB		Up	Diagnosis and prognosis	[150]
Glioma	cir-ITCH	Tissue	Down	Diagnosis and Prognosis	[151]
	circCPA4	Tissue	Up	Prognosis	[91]
Nasopharyngeal carcinoma	circRNA_0000285	Tissue and serum	Up	Prognosis	[152]
Oral squamous cell carcinoma	hsa_circ_001242	Tissue	Down	Diagnosis	[153]
Cholangiocarcinoma	Cdr1as	Tissue	Up	Prognosis	[154]
Pancreatic cancer	circ-LDLRAD3	Tissue and plasma	Up	Diagnosis	[155]
Papillary thyroid carcinoma	hsa_circ_0137287	Tissue	Down	Diagnosis	[156]
Cervical cancer	circRNA8924	Tissue	Up	Diagnosis	[157]

## Conclusion

In this review, we briefly summarized the characteristics, biogenesis, classification, and mechanisms of circRNAs and their relationship with cancer. CircRNAs are stable, highly conserved and cell- and tissue-specific molecules in the RNA interaction network, indicating that they are not erroneous or random byproducts but are derived from a strictly controlled biological process. Currently, research on circRNAs mainly focuses on their expression pattern in cancer and their biological role in tumorigenesis. CircRNAs can function as miRNA sponges, which is by far the most studied mechanism. In addition, circRNAs can regulate gene expression at the transcriptional and posttranscriptional levels and can be translated into proteins. Among mechanistic studies, miRNA sponges are the most studied; thus, more attention should be paid to the exploration of gene regulation and translation mechanisms of circRNAs in the future.

It has been reported that the close relationship between the level of circRNAs and clinical features, such as tumor size, TNM stage and lymphatic metastasis, and circRNAs could be reflected in extracellular fluid through exosomes, and the steady expression of circRNAs in plasma [104] indicates that circRNAs can be biomarkers of the diagnosis and prognosis of cancer.

Due to the steady development of RNA technology, we expect that the field of circRNAs will have considerable development in the future. The exact location, transport, degradation of circRNAs in living cells, and the complete circRNAs interactome will all make progress in this field.

## Data Availability

The datasets used and/or analyzed during the current study are available from the corresponding author on reasonable request.

## Abbreviations

lncRNAs: Long noncoding RNAs
miRNAs: MicroRNAs
circRNAs: Circular RNAs
DCC: Deleted in colorectal cancer
RNA-seq: RNA sequencing
pre-mRNAs: Precursor messenger RNAs
RBPs: RNA binding proteins
ADAR: Adenosine deaminase
SFs: Splicing factors
ecircRNA: CircRNA from exons
ciRNA: CircRNA from introns
EIciRNA: CircRNA from exon–intron junctions
MREs: MiRNA response elements
MSC: Mesenchymal stem cell
m^7^G: 7-methylguanosine
ORF: Open reading frame
GFP: Green fluorescent protein
IRESs: Internal ribosome entry sites
m^6^A: N6-methyladenosine
snRNP: Small nuclear ribonucleoprotein
Pol II: Polymerase II
MBL: Muscleblind
TME: Tumor microenvironment
ECM: Extracellular matrix
HCC: Hepatocellular cancer
ceRNA: Competing endogenous RNA
PVTT: Portal vein tumor thrombus
EMT: Mesenchymal transition
TNBC: Triple-negative breast cancer
DFS: Disease-free survival
OS: Overall survival
TAM: Tamoxifen
GC: Gastric cancer
CDDP: Cisplatin
LUSC: Lung squamous cell carcinoma
PCNA: Proliferating cell nuclear antigen
CRC: Colorectal cancer
AML: Acute myeloid leukemia
ESCC: Esophageal squamous cell carcinoma
ACT: Analysis of common targets
